# Sex differences in the relationship between parathyroid hormone and uric acid in osteoporotic fracture patients: insights from a retrospective cross-sectional study

**DOI:** 10.3389/fendo.2025.1621971

**Published:** 2025-10-15

**Authors:** Cheng-bai Zhu, Peng Zhou, Ke Lu, Chong Li, Yin-lin Wei, Jian Jin, Wen-bin Hu, Yi-jun Gao

**Affiliations:** ^1^ Department of Orthopedics, Affiliated Kunshan Hospital of Jiangsu University, Suzhou, Jiangsu, China; ^2^ Kunshan Municipal Health and Family Planning Information Center, Suzhou, Jiangsu, China; ^3^ Chronic Disease Department, Kunshan Center for Disease Control and Prevention, Suzhou, Jiangsu, China

**Keywords:** osteoporotic fracture, parathyroid hormone, uric acid, sex differences, BMD

## Abstract

**Background:**

Osteoporosis (OP) is characterized by decreased bone mineral density and increased fracture risk, particularly in older adults. The relationship between parathyroid hormone (PTH) and uric acid (UA) levels among osteoporotic fracture (OPF) patients remains unclear. This study aimed to investigate the association between PTH and UA in a large OPF patient cohort.

**Methods:**

In this retrospective cross-sectional study, clinical data from 1,730 OPF patients admitted to Kunshan Hospital of Jiangsu University between January 2017 and August 2023 were analyzed. Baseline PTH and UA levels were measured, and analyses adjusted for age, sex, body mass index (BMI), and other clinical parameters. Multivariable logistic regression, smooth curve fitting, and threshold analyses were conducted.

**Results:**

After stratifying by gender and adjusting for covariates, regression analysis revealed a significant positive association between PTH and UA in males, with each unit increase in PTH corresponding to a 2.19 µmol/L rise in UA (β=2.19, 95% CI: 1.27–3.12, p<0.01). Similarly, females exhibited a positive association, with each unit increase in PTH associated with a 0.88 µmol/L increase in UA (β=0.88, 95% CI: 0.35–1.40, p<0.01). Additionally, a nonlinear relationship was identified in female patients, with a UA inflection point at 26.14 µmol/L.

**Conclusion:**

A significant positive correlation between PTH and UA levels exists among OPF patients, with males exhibiting a linear and females a nonlinear relationship. These findings highlight the importance of gender-specific personalized management strategies in clinical practice.

## 1 Introduction

Osteoporosis (OP) is a disease condition whereby the patient experiences a marked reduction in bone density and degradation of bone microstructure, thereby increasing risk of fractures (1, 2). As people age, the risk of OP and related fractures rises drastically, particularly, among older adults. This risk is even more pronounced among women (3). OP diagnosis relies on the bone mineral density (BMD) assessment (4). Osteoporotic fracture (OPF) and related complications are common among women aged 55 and older, as well as men aged 65 and older (5). A reduction in BMD substantially enhances fracture and non-skeletal injury risk, which, in turn, produces subsequent pain and poor quality of life (6). Most OPF patients OPFs also suffer from secondary causes, ranging from endocrine disorders to chronic inflammation and genetic diseases, all of which can potentially accelerate OP progression (7).

Xanthine oxidase (XO) is a key enzyme in uric acid (UA) production. It catalyzes the oxidation of hypoxanthine to xanthine, and then to UA (8). Emerging evidences suggest that the circulating UA concentration is positively linked to bone mass in most adults, including postmenopausal women and the elderly (9). Others report that augmented serum UA levels are robust biomarkers of bone health, suggesting a possible protective role of UA against bone loss (10). Elevated levels of UA may help maintain higher bone mineral density, potentially through its antioxidant properties, which may inhibit osteoclast-mediated bone resorption (11). Conversely, some studies do not support the beneficial effects of UA on bone metabolism (11).

Parathyroid hormone (PTH) simultaneously enhances both bone resorption and bone formation (12), However, its net effect depends on the frequency of exposure (13). Owing to its dual effect on bone remodeling (14), it is also a key regulator of bone metabolism. PTH is secreted by the chief cells of the parathyroid gland, and it influences calcium and phosphorus homeostasis via interaction with specific receptors in critical tissues, such as, bone and kidneys (12).

The relationship between PTH and UA contents among OP patients is not fully understood. Some evidences suggest a positive correlation between the two, whereby PTH promotes bone resorption while indirectly activating osteoblasts (15). Till date, there are limited studies exploring the relationship between PTH and UA content in OP. Clarifying this relationship can enhance our understanding of bone metabolism and improve clinical management of OP patients (16, 17). In epidemiological analyses of OPF patients, OPF patients are significantly more prevalent among female versus male patients. This marked gender disparity prompts our investigation into the sex-specific influences on PTH and UA levels. Drawing from these epidemiological data, we hypothesize that sex-specific variations are present among OPF patients. Therefore, this study aims to investigate the association between PTH and UA contents, with particular emphasis on elucidating sex-specific patterns in this relationship.

## 2 Materials and methods

### 2.1 Ethical statement

This study received approval from the Ethics Committee of the affiliated Kunshan Hospital of Jiangsu University, Suzhou, China (approval No. 2024-03-053-H00-K01), and closely followed the principles outlined in the Declaration of Helsinki. Patient identities were concealed to ensure an unbiased investigation. All patients provided written informed consent before participation in the study.

### 2.2 Study design and patient clinical cohorts

**Figure 1:**
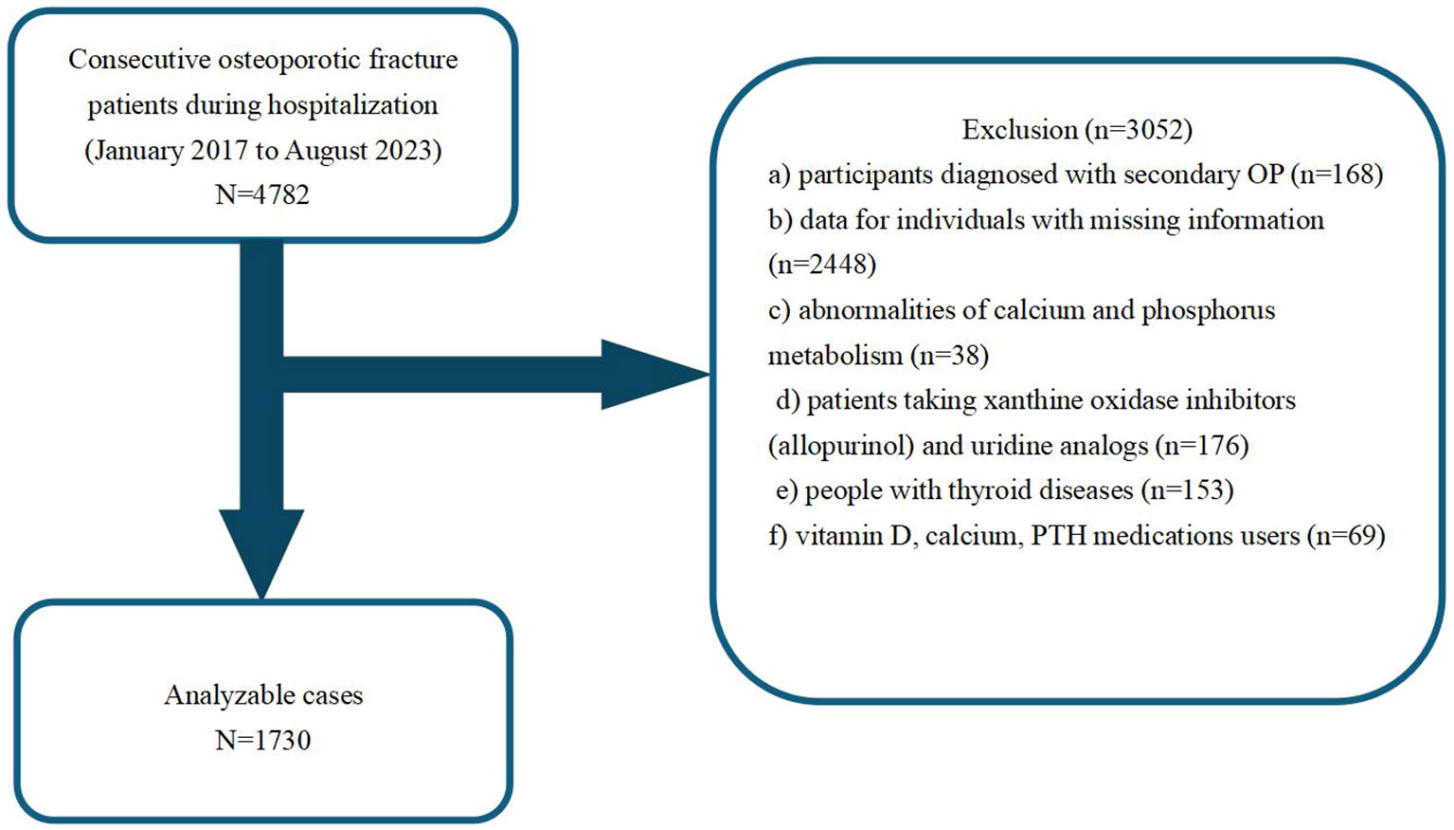
A schematic representation of our study design.

This retrospective cross-sectional study collected patient medical data between January 2017 and August 2023 from the Kunshan Hospital, affiliated with Jiangsu University, Suzhou, China. Our analysis initially included a cohort of 4782 OPF patients, who received surgical inpatient treatment or required hospitalization at the participating institution (18). Individuals with the following criteria were not eligible for analysis: those with a) secondary OP diagnosis (n =168); b) missing medical information (n =2448); c) abnormalities in calcium and phosphorus metabolism (n =38); d) consumers of xanthine oxidase inhibitors (allopurinol) and uridine analogs (n =176); e) diagnosed with thyroid diseases (n =153); f) vitamin D, calcium, and PTH medications users (n =69). OP diagnosis was made when fragility fractures were present, and in absence of other metabolic bone diseases, even when BMD was normal. In the absence of fractures, a T-score ≤ -2.5 was considered to be OP (19). Using the above inclusion and exclusion criteria, we ultimately included 1730 OPF inpatients in the final analysis. **Figure 1** summarizes our strict patient screening process.

### 2.3 Exposure and outcome variables

The endpoint variable was UA concentration. Patient UA content was measured using the automated enzymatic reaction and optical detection techniques of the Beckman AU5800 biochemical analyzer. All measurements were conducted by the same experienced operator and utilized the same instrument.

The independent variable was the fasting circulating PTH levels gathered during the early hours of the day, measured using the chemiluminescence technique on the Beckman Coulter Unicel DXI 800 instrument (Beckman Coulter Inc., Brea, CA, USA), employing the resistivity method.

### 2.4 Covariate variables

Covariates were defined as patient age, gender, body mass index (BMI), Serum calcium, Serum creatinine (CR), Serum UA, Serum urea nitrogen (UN), alcohol consumption, serum phosphorus, smoking status, hypertension, diabetes, and fracture category. Frequent alcohol consumers were defined as those who consumed alcohol weekly for the past 12 months (18). Smokers were defined as those who actively smoked or had smoked previously during the past 12 months (18). The examined fracture types were in the following locations: wrist, thoracic vertebrae, lumbar vertebrae, and femoral trochanters, proximal humerus. All clinical parameters were assessed while patients were in a fasting state within three days of their hospital admission.

### 2.5 Statistics

Data pertaining to patient demographics, laboratory tests, and clinical outcomes are presented as medians or means ± standard deviation (SD) within the interquartile range (25th and 75th percentiles). The data for each category are expressed as frequency (or percentages). Categorical data analysis was performed using the Pearson’s chi-square or Fisher’s exact test for univariate analysis. Normally distributed continuous data were analyzed using the independent samples t-test, while the non-normally distributed continuous data were evaluated using the Mann-Whitney U test.

Generalized estimating equations (GEE) and generalized additive models (GAM) are two frequently used statistical modeling methods (20, 21). GEE models the average response and correlation by specifying a working relation structure appropriate for handling correlated data, namely, longitudinal or clustered data. In contrast, GAM utilizes flexible non-parametric smoothing functions to examine intricate nonlinear relationships between the response and predictors without assuming a parametric form. Both methods require response distribution specification, formulation of a mean model, and usage of iterative algorithms for parameter estimation. Users can then determine model fit and perform subsequent statistical inference.

Using GEE, we next appropriately adjusted for covariates and examined the independent relationship between PTH and UA contents in OPF patients. The developed models included both unadjusted and slightly adjusted models, referred to as Model 1 and Model 2, respectively, as well as a fully adjusted model, which was Model 3. We also conducted variance inflation factor (VIF) analysis to detect multicollinearity among the covariates.

Thereafter, the variables were adjusted according to the following criteria: (1) a significant alteration of at least 10% in the odds ratio (OR) when including or excluding covariates in the baseline or complete model; (2) covariates that met criterion 1 or exhibited a p-value < 0.1 in univariate models. Models 2 and 3 were adjusted for covariates using criteria 1 and 2, respectively. Finally, the three aforementioned models were generated as follows: Model 1 was unadjusted; Model 2 (the minimally adjusted model) considered age, and BMI; and Model 3 included patient age, BMI, UA, UN, CR, serum calcium, serum phosphorus, diabetes, hypertension, alcohol consumption, smoking status, and fracture category.

All statistical analyses utilized the R software version 3.6.3 (http://www.r-project.org) and Empower Stats (www.empowerstats.com, X&Y Solutions, Inc., Boston, MA, USA), and significance was adjusted to a p-value of 0.05 or below.

## 3 Results

### 3.1 Clinical and demographic characteristics of study participants based on gender

Using the eligibility criteria detailed in **Figure 1**, we recruited 1,730 patients treated between January 2017 and August 2023 for analysis. **Table 1** summarizes their characteristics based on gender. The study cohort consisted of 73.99% females (n=1280) and 26.01% males (n=450). The mean participant age was similar across both genders, with females at 69.61 ± 10.57 years and males at 69.07 ± 11.80 years. We observed notable differences in smoking and alcohol consumption between males and females, wherein a substantially higher percentage of males reported smoking (18.00%) and alcohol consumption (10.22%), in relation to females (0.31% and 0.08%, respectively). The circulating calcium and phosphorus concentrations were comparable between the sexes, however, there were slight variations in other parameters, such as, serum creatinine and parathyroid hormone contents. Overall, our data indicated distinct gender differences in lifestyle factors and certain biochemical markers, which may have implications for health outcomes and medical interventions.

**Table 1:** Characteristics of study participants based on different genders.

Sex	Female Mean ± SD	Male Mean ± SD	P-value	P-value*
Age, years	69.61 ± 10.57	69.07 ± 11.80	0.37	0.20
BMI, kg/m^2^	23.35 ± 3.29	22.81 ± 3.17	<0.01	0.01
Serum calcium, mmol/L	2.21 ± 0.13	2.20 ± 0.13	0.07	0.18
Serum Phosphorus, mmol/L	1.06 ± 0.21	1.07 ± 0.21	0.24	0.14
Serum UN, mmol/L	6.13 ± 4.51	6.07 ± 2.57	0.79	0.92
Serum UA, µmoI/L	282.85 ± 92.19	282.85 ± 92.50	0.99	0.91
PTH, pg/mL	13.11 ± 8.71	12.81 ± 8.64	0.53	0.30
Serum CR, µmoI/L	65.54 ± 28.95	66.75 ± 43.72	0.51	0.74
Alcohol consumption, N (%)			<0.01	Reference
No	1279 (99.92%)	404 (89.78%)		
Yes	1 (0.08%)	46 (10.22%)		
Smoking status, N (%)			<0.01	Reference
No	1276 (99.69%)	369 (82.00%)		
Yes	4 (0.31%)	81 (18.00%)		
Diabetes, N (%)			0.83	Reference
No	1220 (95.31%)	430 (95.56%)		
Yes	60 (4.69%)	20 (4.44%)		
Hypertension, N (%)			0.58	Reference
No	1081 (84.45%)	385 (85.56%)		
Yes	199 (15.55%)	65 (14.44%)		
Fracture category, N (%)			<0.01	Reference
Thoracic vertebra	238 (18.59%)	51 (11.33%)		
Lumbar vertebra	388 (30.31%)	123 (27.33%)		
Wrist	69 (5.39%)	16 (3.56%)		
Proximal humerus	180 (14.06%)	59 (13.11%)		
Femoral trochanteric	405 (31.65%)	201 (44.67%)		

*Kruskal-Wallis rank test for continuous variables, Fisher exact for categorical variables with expects<10.

BMI, body mass index; Serum CR, Serum creatinine; Serum UA, Serum uric acid; Serum UN, Serum urea nitrogen; PTH, parathyroid hormone.

### 3.2 Univariate analysis of UA based on gender characteristics

Univariate analysis was conducted to explore the relationship between PTH and UA contents, stratified by gender (**Table 2**). Among females, age exhibited a negative correlation with UA content, with a β coefficient of -0.24 (95% CI: -0.72, 0.23; p=0.32). Alternately, among males, there was a positive correlation with age, yielding a β coefficient of 0.72 (95% CI: -0.00, 1.44; p=0.05). Circulating calcium concentration revealed a marked positive association with UA levels in both females (β = 104.86; p<0.01) and males (β = 89.76; p=0.01). Other variables, such as, BMI and serum phosphorus content, did not demonstrate significant relationships with UA content in either gender. Of note, both genders exhibited marked positive associations between UA and UN contents (females: β = 4.54; p<0.01; males: β = 12.12; p<0.01), as well as between serum UA and serum creatinine levels (females: β = 1.41; p<0.01; males: β = 0.63; p<0.01). In addition, the smoking status, alcohol consumption, diabetes, hypertension, and fracture category did not reveal strong correlations with UA in either gender. Based on these findings, the association between PTH and UA contents may differ by gender, emphasizing the need for tailored approaches during clinical assessments.

**Table 2:** Univariate analysis of UA based on different genders.

Sex	Female	Male
	UA	UA
	β^a^(95%CI)	β^a^(95%CI)
	*P*-value	*P*-value
Age, years	-0.24 (-0.72, 0.23) 0.32	0.72 (-0.00, 1.44) 0.05
BMI, kg/m^2^	-0.39 (-1.92, 1.15) 0.62	-1.01 (-3.71, 1.68) 0.46
Serum calcium, mmol/L	104.86 (66.35, 143.37) <0.01	89.76 (25.18, 154.34) 0.01
Serum Phosphorus, mmol/L	7.31 (-16.24, 30.86) 0.54	23.63 (-18.00, 65.25) 0.27
Serum UN, mmol/L	4.54 (3.45, 5.64) <0.01	12.12 (8.98, 15.26) <0.01
Serum CR, µmoI/L	1.41 (1.26, 1.57) <0.01	0.63 (0.45, 0.82) <0.01
PTH, pg/mL	2.90 (1.95, 3.86) <0.01	2.90 (1.95, 3.86) <0.01
Smoking status, N (%)		
No	Reference	Reference
Yes	55.32 (-35.15, 145.80) 0.23	1.24 (-21.03, 23.51) 0.91
Alcohol consumption, N (%)		
No	Reference	Reference
Yes	115.24 (-65.49, 295.97) 0.21	20.83 (-7.35, 49.00) 0.15
Diabetes, N (%)		
No	Reference	Reference
Yes	16.73 (-7.15, 40.62) 0.17	-5.49 (-47.01, 36.03) 0.80
Hypertension, N (%)		
No	Reference	Reference
Yes	0.72 (-13.23, 14.66) 0.92	1.24 (-23.10, 25.58) 0.92
Fracture category, N (%)		
Thoracic vertebra	Reference	Reference
Lumbar vertebra	4.04 (-10.85, 18.92) 0.60	-7.78 (-38.03, 22.46) 0.61
Wrist	4.87 (-19.85, 29.58) 0.70	-27.13 (-79.18, 24.91) 0.31
Proximal humerus	3.82 (-14.04, 21.68) 0.68	-9.81 (-44.53, 24.92) 0.58
Femoral trochanteric	-6.19 (-20.95, 8.58) 0.41	2.30 (-26.17, 30.78) 0.87

a The dependent variable was UA and β is the result of univariate analysis for UA.

CI, confidence interval; BMI, body mass index; Serum CR, Serum creatinine; Serum UA, Serum uric acid; Serum UN, Serum urea nitrogen; PTH, parathyroid hormone.

### 3.3 Sex-stratified analysis of the association between PTH and UA concentrations

We employed three models to evaluate the correlation between PTH and UA, stratified by gender (**Table 3**). In Model 1, no adjustments were made, and it revealed a strong positive link between PTH and UA in both females (β=1.87, 95% CI: 1.30 to 2.44, p<0.01) and males (β=2.90, 95% CI: 1.95 to 3.86, p<0.01). In Model 2, we adjusted for age and BMI, and the results remained consistent, with PTH displaying marked associations (females: β=1.87, 95% CI: 1.30 to 2.44, p<0.01; males: β=2.84, 95% CI: 1.88 to 3.80, p<0.01). In Model 3, further adjustments were made for additional variables, such as, circulating calcium, circulating creatinine, UA, urea nitrogen, alcohol consumption, serum phosphorus, smoking status, hypertension, diabetes, and fracture category. The associations persisted, with PTH significantly correlating with UA levels (females: β=0.88, 95% CI: 0.35 to 1.40, p<0.01; males: β=2.19, 95% CI: 1.27 to 3.12, p<0.01).

**Table 3:** The relationship between parathyroid hormone and UA in different models based on gender.

Sex		Model 1^a^ N=1730		Model 2^b^ N=1730		Model 3^c^ N=1730	
		β (95%CI)	*P* value	β (95%CI)	*P* value	β (95%CI)	*P* value
Female	PTH pg/mL	1.87 (1.30, 2.44)	<0.01	1.87 (1.30, 2.44)	<0.01	0.88 (0.35, 1.40)	<0.01
Male		2.90 (1.95, 3.86)	<0.01	2.84 (1.88, 3.80)	<0.01	2.19 (1.27, 3.12)	<0.01

a No adjustment.

b Adjust for age, BMI.

c Adjust for age, BMI, Serum calcium, Serum CR, Serum UA, Serum UN, alcohol consumption, Phosphorus, smoking status, hypertension, diabetes, fracture category.

CI, confidence interval; BMI, body mass index; Serum CR, Serum creatinine; Serum UA, Serum uric acid; Serum UN, Serum urea nitrogen; PTH, parathyroid hormone.

### 3.4 Gender-based analysis of spline smoothing curves and threshold effects

**Figure 2:**
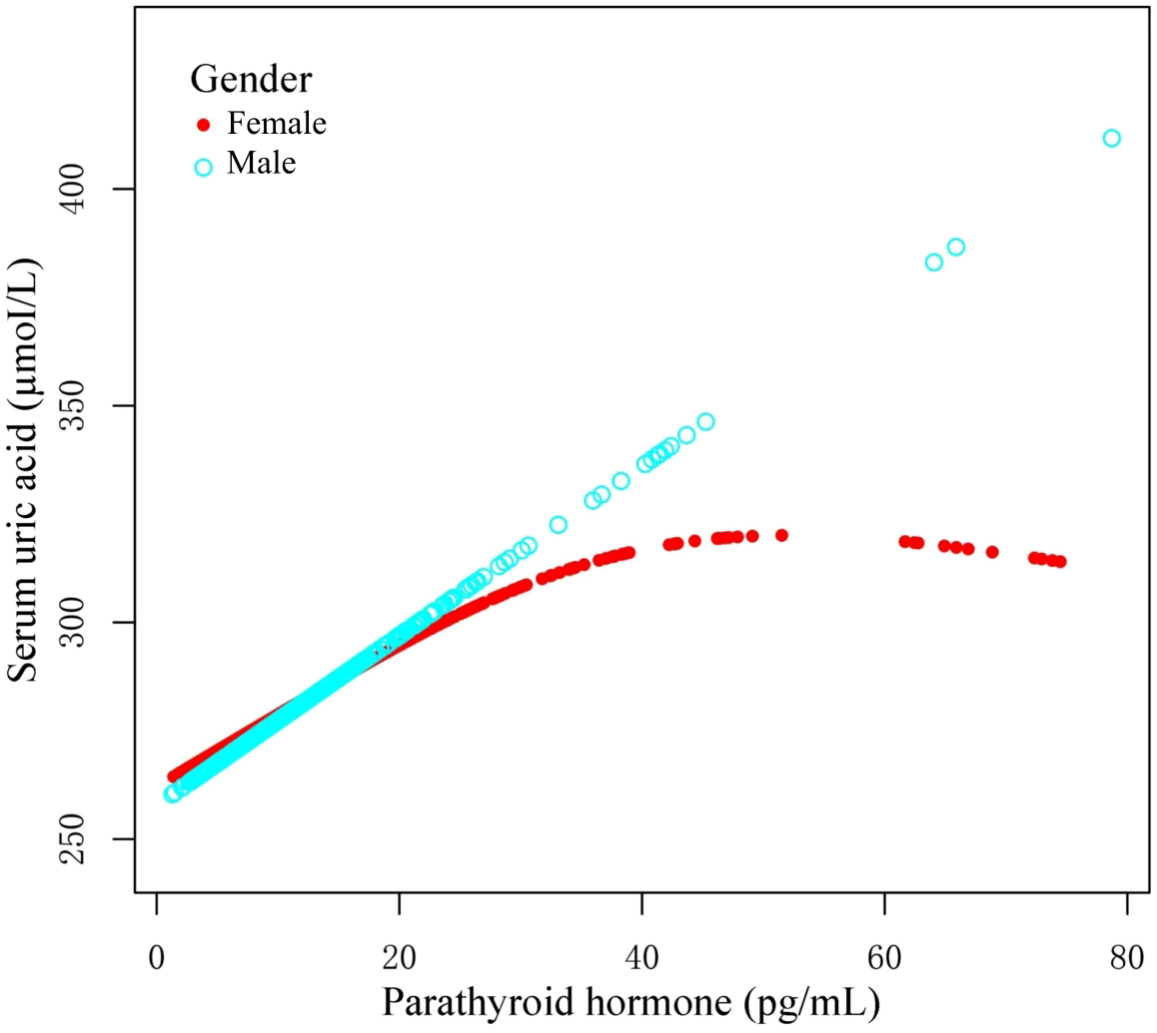
Curves illustrating the relationship between parathyroid hormone (PTH) and uric acid (UA) contents, based on the measured data. The red curve demonstrates the overall trend among females, whereas, the blue curve represents the corresponding trend among males. These trends may indicate a possible gender-specific association between PTH and UA concentrations.

Using graphs, we next evaluated the association between PTH and UA levels between male and female patients, aiming to ascertain the existence of a linear or nonlinear distribution (**Figure 2**). GAM estimation revealed that, following covariates correction, there was a significant linear relationship between PTH and UA contents among the male OPF population (**Table 4**). In addition, we observed a significant nonlinear association between the PTH and UA levels among the female OPF population, modeled using piecewise linear regression using an identified breakpoint (k-value) of 0.03 (**Table 4**).

**Table 4:** Threshold analysis examines the PTH-UA relationship across genders.

Sex	Model 3^a^	Model 3^a^
	Female	Male
	UA β (95% CI)*P*-value	UA β (95% CI)*P*-value
Model A^b^		
One line slope	0.88 (0.36, 1.40) <0.01	2.18 (1.26, 3.11) <0.01
Model B^c^		
PTH turning point (K)	26.14	6.80
<K	1.59 (0.75, 2.42) <0.01	-5.15 (-13.29, 2.98) 0.22
>K	-0.14 (-1.21, 0.93) 0.79	2.56 (1.55, 3.57) <0.01
Slope 2-Slope 1	-1.73 (-3.31, -0.15) 0.03	7.71 (-0.79, 16.22) 0.08
LRT^d^	0.03	0.07

a Adjusted for age, BMI, Serum calcium, Serum CR, Serum UA, Serum UN, alcohol consumption, Phosphorus, smoking status, hypertension, diabetes, fracture category.

b Linear analysis, p value<0.05 indicates a linear relationship.

c Nonlinear analysis.

d *P*-value <0.05 means Model B is not significantly different from Model A, which indicates a nonlinear relationship.

BMI, body mass index; Serum CR, Serum creatinine; Serum UA, Serum uric acid; Serum UN, Serum urea nitrogen; PTH, parathyroid hormone.

In the threshold analysis of PTH and UA levels among male and female OPF patients, no threshold effect was observed for males, attributable to the direct line impact of the linear association. In case of females, the impact size on the left side of the threshold was 1.59 (95% CI: 0.75 to 2.42, p<0.01), and on the right side of the threshold, it was -0.14 (95% CI: -1.21 to -0.98, p=0.79).

## 4 Discussion

This cross-sectional investigation analyzed the relationship between PTH and UA contents among OPF patients, and demonstrated a discreet sex-specific pattern. Among men, elevated PTH content linearly correlated with augmented serum UA levels, whereas among women, the PTH–UA relationship was nonlinear, with an inflection around 26.14 pg/mL PTH. Beyond this threshold, the UA content among women plateaued despite further increases in PTH content. These findings suggest that upregulated PTH levels may confer a protective effect on UA levels among female OPF patients, an effect that is absent among males under similar conditions. This sex difference in the PTH–UA association is a novel observation, and has potential clinical significance in OP management.

Our results support prior evidence of an independent relationship between serum PTH and UA contents (14). Earlier population-based studies (largely using mixed or male-dominant cohorts) have consistently reported that individuals with higher PTH also possess enhanced UA levels, and, therefore, experience greater odds of hyperuricemia (14). For instance, a nationally representative U.S. survey revealed that the circulating UA levels increased in proportion with PTH concentration, even after adjustments for age, renal function, and other factors. Likewise, baseline data from studies involving men with advanced age demonstrated a positive PTH–UA relationship (14). These earlier investigations, however, did not specifically focus on osteoporotic patients or thoroughly examine sex differences. Our study addresses this gap by demonstrating that the PTH–UA link holds true in a high-risk osteoporotic fracture population and by highlighting a clear divergence between males and females (14, 22). In doing so, we provide novel insight into the sex-specific mechanisms that earlier general population studies have only speculated. Moreover, hypoparathyroidism has been associated with increased fracture risk – particularly vertebral fractures, despite presence of often normal or even high BMD (23, 24). Teriparatide is reported to reduce fracture incidence among severe OP patients (3). Our findings build on this paradox by suggesting that among women with OP, a moderate PTH elevation may be beneficial via its impact on UA, whereas among men, the PTH benefit on UA appears linear and perhaps more limited. In terms of bone health, these findings coincide with known influences of PTH. Intermittent PTH administration exerts anabolic effects on bone, increasing bone formation and density. Conversely, primary or secondary hyperparathyroidism preferentially promotes cortical bone loss and increases fracture risk (25). Hence, an optimal PTH range is critical for skeletal integrity. This study suggests that, within that range, the interaction with UA differs by sex (24). The demonstrated independent association between PTH and UA among OPF patients corroborates earlier reports in other populations, reinforcing that the PTH–UA relationship is a robust phenomenon (14, 24). Moreover, by stratifying the analysis by gender, we further provided evidence that the nature of this relationship is modified by sex, which earlier works hinted upon (14).

Overall, our results both confirmed the general positive link between PTH and UA contents noted in prior studies, and expanded the existing literature by characterizing the relationship divergence among men and women in a clinical high-risk population. There is strong support for a causal effect of PTH on UA content from clinical trials examining teriparatide. In a large fracture prevention trial, teriparatide therapy proportionately increased hyperuricemia incidence, in a dose-dependent manner (26). Teriparatide treatment induced marked rises in serum UA levels, compared to placebo, particularly at the higher dosage and among patients with reduced renal function. Notably, UA concentration fell back down after teriparatide discontinuation, confirming a reversible PTH-driven influence on urate homeostasis (26, 27). Although teriparatide significantly increased UA content, clinical gout or urate crystal events did not rise in the aforementioned trials. These pharmacologic data substantiate that PTH elevations can directly increase UA levels. Mechanistically, PTH may reduce the fractional urate excretion by kidneys (14, 22). The proximal tubule reabsorbs a majority of filtered urate via transporters, such as, URAT1 (SLC22A12) and GLUT9 (SLC2A9), using processes that are modulated by sodium balance and hormonal signals (14). The co-regulation of sodium and urate reabsorption potentially hints towards an indirect PTH-sodium transport-mediated regulation of urate reuptake. Overall, the net PTH effect appears to favor urate retention, clarifying the positive PTH–UA relationship seen in epidemiologic studies and in our cohort (14).

Interestingly, UA effects are biphasic. While moderate UA elevations may benefit bone, excessive UA is often detrimental (16, 28). Extremely high UA content can precipitate as crystals and incite inflammation. Moreover, chronic hyperuricemia is a known risk factor for gout, nephrolithiasis, and cardiovascular disease (29). Emerging reports reveal a U-shaped association between UA and bone health: both low and very high UA levels may increase OP risk (29). For instance, one study involving hypertensive older adults demonstrated that the skeletal benefits of UA plateau and then reverse at very high UA concentrations, with both the lowest and highest UA tertiles showing enhanced fracture risk, relative to mid-range UA (30). Hence, while PTH-induced UA elevation may be favorable to a certain point, an excess rise in UA may confer no further bone benefit and may even pose systemic risks (31). This nuance may underlie the plateau we observed among women: once PTH reached a certain level, additional PTH did not further raise the UA concentration, perhaps due to the limitations in our physiological measures, such as urate precipitation or maximum reabsorption capacity being reached. In summary, the PTH impact on UA concentration and the subsequent effects on bone are a complex interplay.

Sex-based differences are a central finding of this study. We observed a nonlinear PTH–UA association among women, and a linear association among men (32). Several biological factors likely contributed to this disparity (21). For instance, the influence of sex hormones on urate metabolism. It is well documented that, prior to menopause, women possess drastically lower serum UA levels, compared to men. Estrogen accelerates urate clearance by augmenting renal urate excretion; and simultaneously, it downregulates urate reabsorption transporters within the kidney, which, in turn, results in a reduced UA set-point among women (33). Population studies show that hyperuricemia is far less prevalent in premenopausal women than in men (34). Following menopause, the UA content rises substantially among women, approaching those of men, and hormone replacement therapy attenuates this rise (33). These observations implicate estrogen as a protective agent against urate accumulation. Among our female patients, most of whom fall under the postmenopausal category, diminished estrogen activity likely facilitated the PTH-induced urate retention, but maybe only up to a certain extent (30). We observed in women likely reflects a balance between PTH-driven urate retention at lower PTH ranges and counter-regulatory ceiling effects at higher PTH, whereas men—lacking estrogen-mediated uricosuria—exhibit a more linear coupling. We speculate that at lower PTH ranges, rises in PTH potentially overrides the baseline uricosuric effects, causing UA to climb rapidly.

The clinical consequences of the aforementioned sex difference are noteworthy. Since premenopausal women naturally exhibit reduced UA levels, they may rely less on the UA-mediated bone-protective effects, as their estrogen provides direct skeletal protection (35). However, following menopause, women lose the estrogen-based protective effect on bone, and the estrogen’s uricosuric effect causes women’s UA levels to remain relatively lower than men’s (36). This places postmenopausal women at a double disadvantage: higher bone resorption due to estrogen loss, and reduced antioxidant protection from UA (37). In this context, our finding that augmented PTH among women raises UA levels suggests a compensatory mechanism that may mitigate bone loss (38). Essentially, the PTH tendency to raise UA content may be more beneficial among women because it counteracts, in part, the low-UA milieu caused by estrogen deficiency. However, since women are unable to raise UA indefinitely, there may be a limitation to this benefit (39). It also raises a caution: therapeutic suppression of PTH among postmenopausal women may inadvertently lower UA levels, and remove some of its bone-protective antioxidant influence. Of course, this hypothesis requires further exploration, but it underscores the interconnected nature of endocrine regulation among women (10).

Overall, the pronounced sex differences observed in this study emphasize that the hormonal milieu modulates the PTH–UA relationship (9, 38). Estrogen appears to buffer women against both elevated UA levels and high bone turnover, however, once that buffer is removed, women exhibit a unique PTH–UA pattern that likely reflects an equilibrium between the beneficial and adverse impacts of PTH (24, 30). Men, lacking this estrogen-mediated action, exhibit a more direct PTH–UA coupling and therefore a distinct risk-benefit balance (40). These insights highlight the significance of considering patient sex while interpreting PTH and UA levels during management of OP and other bone metabolic diseases. In summary, this study’s insights advocate for a more nuanced approach to bone metabolic disease, factoring in sex hormones and urate metabolism. Understanding these interactions will ultimately contribute to more tailored OP care approach.

This study benefits from a large OPF patients sample size, as well as a comprehensive dataset collected over several years. Our focus on a homogenous high-risk patient population enhances the clinical relevance of our findings to patients most in need of secondary fracture prevention. Unlike numerous prior investigations, we performed a sex-stratified analysis, which uncovered the nuanced differences in the PTH–UA relationship between men and women. The use of rigorous statistical methods is a definite strength, as it allowed for the identification of the inflection point among women. Additionally, all biochemical measurements were conducted in a controlled hospital laboratory setting, which likely reduced measurement variability.

We also acknowledge several limitations. First, the cross-sectional nature of this study precludes causal inferences. We cannot definitively establish that a rise in PTH levels causes alterations in the UA content; the association may be bidirectional or influenced by an unmeasured factor. Although we adjusted for renal function and other variables, residual confounding is possible – notably, we were unable to gather data on diuretic usage or purine intake, which can affect UA concentration. Second, the PTH and UA contents were measured at a single time point upon admission. Acute fracture- or surgery-related alterations can potentially influence both PTH and UA levels. Thus, our proposed mechanism of UA-based antioxidant protection among women remains speculative. Third, our study population was relatively homogeneous, comprising only data from patients with osteoporotic fractures, which limits generalizability due to the absence of data from healthy individuals. Additional investigations are warranted, particularly those involving biomarkers that can help verify the physiological pathways.

## 5 Conclusion

In conclusion, our findings demonstrate that the PTH-mediated regulation of UA levels serves as an independent protective factor for clinical outcomes among female OP patients, whereas this protective effect is not present among male patients. Additional explorations are warranted to elucidate the underlying mechanisms of this sex-specific difference, and to develop targeted therapeutic interventions that enhance clinical outcomes among OPF patients.

## Data Availability

The original contributions presented in the study are included in the article/supplementary material. Further inquiries can be directed to the corresponding author.
